# Jingfang granules inhibiting LPS-induced acute lung injury via regulating linoleic acid and arachidonic acid metabolism pathway

**DOI:** 10.1371/journal.pone.0340858

**Published:** 2026-01-16

**Authors:** Yuqi Fu, Lei Liu, Guangli Yan, Le Yang, Guimin Zhang, Ling Kong, Yu Guan, Hui Sun, Yongxia Guan, Chang Liu, Ye Sun, Ying Han, Xijun Wang

**Affiliations:** 1 State key Laboratory of Integration and Innovation of Classic Formula and Modern Chinese Medicine, National Chinmedomics Research Center, National TCM Key Laboratory of Serum Pharmacochemistry, Metabolomics Laboratory, Department of Pharmaceutical Analysis, Heilongjiang University of Chinese Medicine, Harbin, China; 2 State Key Laboratory of Dampness Syndrome, The Second Affiliated Hospital Guangzhou University of Chinese Medicine, Guangzhou, China; 3 State key Laboratory of Integration and Innovation of Classic Formula and Modern Chinese Medicine, Lunan Pharmaceutical Group Co. Ltd., Linyi, China; Central South University, CHINA

## Abstract

Acute lung injury (ALI) is a severe clinical syndrome with high mortality. Jingfang Granules (JFG), a modern formulation of the traditional Chinese medicine (TCM) compound Jingfang Baidu Powder, has been widely used to treat ALI. However, its protective effects and underlying mechanisms in ALI remain poorly understood. This study is based on a lipopolysaccharide (LPS)-induced ALI rat model, which was intervened with low, medium, and high dose of JFG. We carried out metabolomic analysis and identified 12 blood metabolites, the levels of core metabolites were regulated under JFG intervention, including L-Carnitine, Citric acid, Taurocholic acid, Arachidonic acid (AA), and Linoleic Acid (LA). Besides blood metabolites, 11 urine metabolites were also callback under JFG intervention, including Valine, Citric acid, L-Phenyalalanine, and Leukotriene B4, mainly involving the LA metabolism, AA metabolism, and phenylalanine, tyrosine and tryptophan biosynthesis. Comprehensive analysis shows that the restored enrichment pathways are mainly concentrated in inflammatory response, amino acid metabolism, and fatty acid metabolism. These findings reveal the potential mechanism of JFG in LPS-induced ALI, and its pathway nodes facilitate rapid translation from laboratory to clinical applications.

## 1. Introduction

ALI is a serious clinical syndrome characterized by damage to alveolar epithelial cells and capillary endothelial cells, leading to diffuse interstitial and alveolar edema, which in turn causes acute hypoxic respiratory dysfunction [1,2]. Severe ALI can develop into acute respiratory distress syndrome (ARDS), which has an extremely high mortality rate and poses a serious threat to the quality of patient’s life [3,4]. At present, the treatment of ALI mainly includes infection control [5,6], prone position [7,8,9], and mechanical ventilation support [10,11]. For many years, the treatment effect has not made breakthrough progress, and the global mortality rate of ALI/ARDS remains high [12,13]. Additionally, the current treatment strategy for ALI is relatively single and lacks diversified methods. Although some drugs such as statins [14,15], anti oxidative stress drugs [16,17], and nonsteroidal anti-inflammatory drugs [18,19] have made some progress in treating ALI, these drugs may also bring side effects while inhibiting the inflammatory response of ALI [20,21,22], and their efficacy and safety still need further research and verification.

Recently, significant progress has been made in the treatment of ALI with TCM. Some Chinese herbal formulas and preparations, including JFG, Huanglian Jiedu Decoction, Gegen Qinlian Decoction, Xuebijing Injection, Jingjie-Fangfeng Couplet, and Jiuwei Qianghuo Decoction, have shown therapeutic effects on ALI in a multi-target and multi-pathway manner by reducing the activation and aggregation of inflammatory cells [23,24], inhibiting inflammatory signaling pathways [25,26], alleviating pulmonary edema [27,28], reducing oxidative stress [29,30], and energy metabolism and immune regulation [31,32]. Notablely, JFG has gained attention for its promising performance in treating ALI, which contains 11 TCM: *Schizonepeta tenuifolia Briq.*, *Saposhnikovia divaricata (Turcz.) Schischk.*, *Heracleum hemsleyanum Diels*, *Notopterygium incisum Ting ex H. T. Chang*, *Bupleurum chinense DC.*, *Peucedanum praeruptorum Dunn*, *Ligusticum chuanxiong hort*, *Poria cocos (Schw.) Wolf*, *Platycodon grandiflorus (Jacq.) A.DC.*, *Citrus aurantium L.*, and *Glycyrrhiza uralensis Fisch.* Their active ingredients are known to inhibit 5-LOX, 12/15-LOX, and COX-2, which are the key gatekeepers of AA and LA metabolism [33]*.* Modern pharmacological studies have shown that its constituent herbs possess potent anti-inflammatory effects, often linked to the inhibition of COX-2 and LOX enzymes, and the modulation of oxidative stress. However, further in-depth research is needed to explore the potential mechanisms of its efficacy and enhance the application of JFG in the treatment of ALI.

Metabolomics, a powerful analytical tool, provides a comprehensive profile of small molecules within biological systems, offering insights into the metabolic pathways and biomarkers associated with disease states and therapeutic interventions [34]. Metabolomics technology is currently one of the most momentous tools for revealing the mechanisms of disease occurrence and potential targets of drug action [35,36]. LC-MS, in particular, has become a cornerstone in metabolomics due to its high sensitivity, resolution, and the ability to identify and quantify a wide range of metabolites [37,38], this unbiased global mining is consistent with the complex intervention mechanism of JFG targeting multiple biological processes simultaneously, making it ideal for discovering both expected and unexpected metabolic changes induced by JFG.

**Fig 1:**
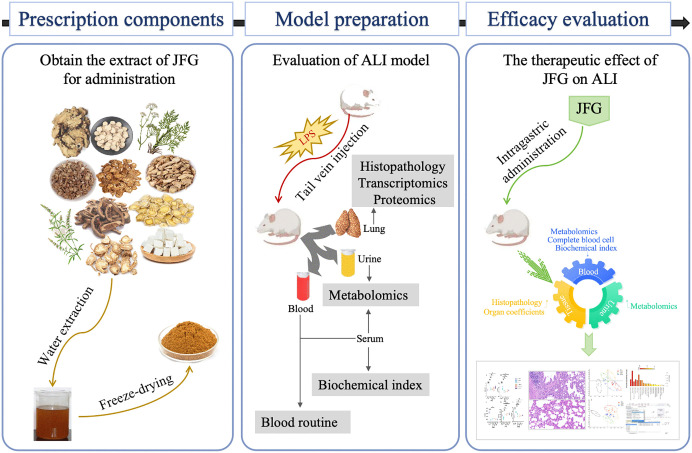
An overview of the experimental design in this study.

Therefore, this study employed an LC/MS-based metabolomics approach to systematically characterize the metabolic alterations in LPS-induced ALI and to identify the key pathways through which JFG exerts its protective effects. By analyzing the metabolic profiles in blood and urine, we identified specific biomarkers and metabolic pathways that are altered in response to JFG treatment, elucidated the biological essence of JFG treatment for ALI, and provided theoretical basis for its further development and widespread application. A detailed overview of the experimental design is shown in Fig 1.

## 2. Materials and methods

### 2.1. Reagents

0.9% NaCl was obtained from Sanlian Pharmaceutical Co., Ltd., (Harbin, China). TNF-*α*, IL-6, IL-1*β*, IFN-*γ*, PGE2, GSH, and MDA ELISA kits were purchased from NanJing JianCheng Bioengineering Institute, (NanJing, China). Leucine enkephalin was obtained from Sigma-Aldrich, (St. Louis, MO, USA). LC-MS and Acquity UPLC HSS T3 Column were purchased from Waters Corporation, (Massachusetts, United States). LPS was obtained from Beijing Solarbio Science & Technology Co.,Ltd., (Beijing, China). The JFG was provided by Lunan Pharmaceutical Group Co., Ltd., (Shandong, China) and the Dexamethasone sodium phosphate injection was purchased from China National Pharmaceutical Group Rongsheng Pharmaceutical Co., Ltd., (Henan, China).

### 2.2. Animals and dosing protocol

Male SD rats weighing 200 ± 20 g were provided by Liaoning Changsheng Biotechnology Co., Ltd., and were maintained at a temperature of 24 ± 2 °C, 12-hour light/dark cycle, and a humidity of 60% ± 10%. Water and food were unlimited to the rats. After adapting for a week, rats were divided into the following six groups randomly and equally: control group (Con, n = 8), positive control group, (DXMS, Dexamethasone sodium phosphate injection, 5 mg/kg/d, n = 8) [39], JFG high-dose group (JFG-H, 4.56 g/kg/d, n = 8), JFG medium-dose group (JFG-M, 2.28 g/kg/d, n = 10), JFG low-dose treatment group (JFG-L, 1.14 g/kg/d, n = 8), and ALI model group (Mod, LPS, 5 mg/kg, n = 8) [40]. The experimental protocol was authorised by the Ethics Committee of Heilongjiang University of Chinese Medicine (2023050602), and all experiments were performed in accordance with the Declaration of Helsinki.

### 2.3. LPS induced ALI model and JFG intervention

After one week of intervention with JFG, LPS solution (5 mg/kg in 0.9% NaCl) was injected into the tail vein of rats in DXMS group, JFG-H group, JFG-M group, JFG-L group, and Mod group. The Con group mice were injected with an equal volume of 0.9% NaCl via the tail vein instead of LPS. After fasting for 24 hours, rats were anesthetized by intraperitoneal injection of 30 mg/kg of 3% pentobarbital sodium by skilled anesthesia and blood collection operators. After confirming that the animals had reached a pain free reflex state, abdominal aortic blood and organ tissues were collected, and finally the animal carcasses were subjected to harmless treatment in accordance with regulations.

### 2.4. Complete blood cell assay and biochemical index detection

A portion of the blood was collected in a sodium citrate blood collection tube for automatic complete blood count within 6 h. The remaining blood was collected using disposable 1.5 mL centrifuge tubes, and centrifuged at 4 °C for 10 minutes at a speed of 4000 r/min for obtaining serum, which was used for measuring biochemical index by ELISA kits, including TNF-*α*, IL-6, IL-1*β*, IFN-*γ*, PGE2, GSH and MDA, which can effectively reflect inflammatory reaction *in vivo*. The remaining serum was stored at −80 °C for subsequent metabolomics experiments.

### 2.5. Analysis of organ coefficients and histopathology

The spleen, lungs, and thymus of rats were removed after blood collection, and then absorbed surface moisture with filter paper after rinsing with 0.9% NaCl. Weighed these organs of rats precisely, using the weight ratio of organ to body as the organ coefficient. Fixed lung tissue in 4% paraformaldehyde, embed it in paraffin, and performed H&E staining analysis to observe the inflammatory infiltration status of the lungs. Histopathology images were captured with a Nikon E200 light microscope.

### 2.6. Untargeted metabolomics

#### 2.6.1. Sample collection and preparation.

After thawing the serum sample in an ice water mixture, an appropriate amount was taken and vortexed it with with methanol. Let it stand for 2 hours and centrifuge at 13000 r/min for 10 minutes at 4 °C to remove protein. The supernatant was used for LC-MS detection.

Placed the rats in a metabolic cage at 7:00 pm on the day of collecting urine samples until 7:00 am the next day. Diluted urine with an equal volume of ultrapure water, vortexed the mixture and then centrifuged at 4 °C and 13000 r/min for 10 minutes. Finally, the supernatant was filtered through a 0.22 *μ*m filter membrane to prepare urine samples for LC-MS detection.

#### 2.6.2. LC-MS analysis.

A Waters ACQUITY UPLC® HSS T3 column was used, and the gradient elution system consists of A (0.1% formic acid in acetonitrile, v/v) and B (0.1% formic acid ultrapure water, v/v) solutions. The elution procedure of serum sample was performed as follows: 0–2 min, 5%−60% A, 2–6 min, 60%−80% A, 6–7 min, 80%−95% A. The column temperature was 40 °C. The flow rate was 0.3 mL/min, and the volume of the injected sample was 3 *μ*L. Differently, the elution gradient of urine sample was: 0–5 min, 1%−10% A, 1–20 min, 20%−50% A, 8–9 min, 50%−90% A. The column temperature was 30 °C. The flow rate was 0.4 mL/min, and the volume of the injected sample volume was 2 *μ*L.

The electrospray ionization ion source temperature was 110 °C, and the desolvation gas flow was 800 L/h in both positive and negative ions modes. The capillary voltage was 3.0 kV and the cone voltage was 30 V in the positive-ion mode while were 2.5 kV and 20 V in the negative-ion mode. The data acquisition rate was set to scan every 0.2s with a scanning delay of 0.1s to collect quality data ranging from 100 to 1200 Da. 400 ng/mL of leucine enkephalin was used for system correction to ensure the accuracy results. These parameters were the same in the detection of serum and urine samples.

#### 2.6.3. Data processing and multivariate statistical analysis.

All LC-MS raw data were preprocessed by Progenesis QI for peak detection, alignment, and deconvolution. Retention time tolerance was set to ± 0.2 min and mass tolerance was set to ± 10 ppm, these were applied to ensure accurate peak matching across samples. Raw spectral data were subjected to baseline correction to remove background noise and instrumental artifacts. A SNR threshold of 10 was applied to eliminate low-intensity signals, and signals below this threshold were excluded from further analysis, aim to effectively minimizing the inclusion of background noise while retaining robust metabolic features for subsequent analysis. Following internal standards-based quality control, total ion current normalization was chosen as our primary method to correct for overall variations in sample concentration and instrument response, and then imported into Ezinfo 3.0 for multivariate statistical analysis using PCA and OPLS-DA. Potential biomarkers were selected based on a combination of multivariate and univariate statistical criteria. VIP > 1, max fold change ≥ 2 and FDR-adjusted *p*-values < 0.05 were confirmed as the initial screening conditions for potential effect markers, which were searched in databases such as HMDB (http://www.hmdb.ca/), KEGG (http://www.kegg.com), and Pubchem (https://pubchem.ncbi.nlm.nih.gov/), and their chemical structures were determined by combining MS/MS data fragment information.

### 2.7. Pathway enrichment and correlation analysis

MetaboAnalyst is a widely recognized powerful analysis tool that greatly integrates the breadth of pathway enrichment analysis and pathway topology analysis, thereby enhancing data visibility. The identified metabolites in metabolomic analysis were subsequently uploaded to the KEGG pathway database, providing profound biological explanations for their underlying functional impacts. A correlation analysis between blood effect biomarkers and biochemical indicators was established using the Chiplot website to screen for key effect markers of JFG against ALI.

### 2.8. Statistical analysis

Difference between multiple groups were assessed by one-way ANOVA or two-way ANOVA. A *p*-value of less than 0.05 was considered statistically significant. The Chiplot website (https://www.chiplot.online/.) and GraphPad Prism 9 software were used for data statistical analysis and visualization.

## 3. Results

### 3.1. Lung pathological damage, change of hematological parameters and increased inflammatory cytokines were found in the ALI model

**Fig 2:**
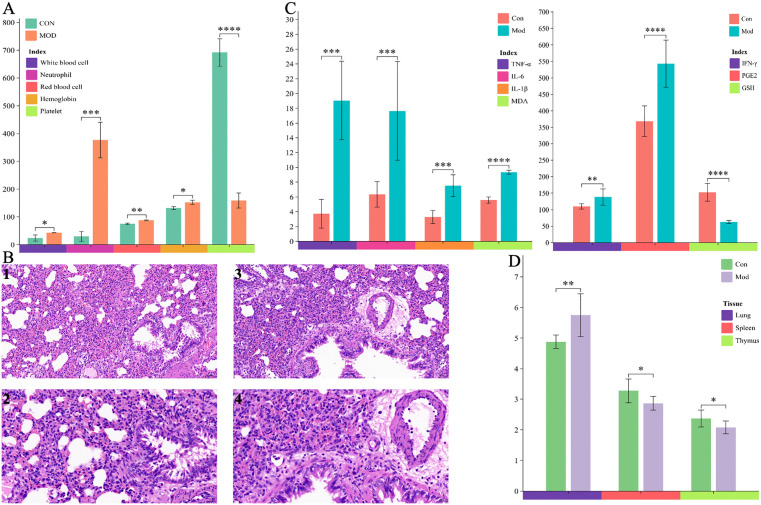
Evaluation of ALI model preparation. **(A)** Number of platelets, white blood cells, and neutrophils in the blood after 24 hours of LPS attack in the control group and ALI model group. **(B)** Hematoxylin and eosin **(H&E)**-stained lung sections of the control group and ALI model group rats at 24h after LPS challenge. **(B-a)** Lungs of blank control group at 20* magnification, **(B-b)** Lungs of blank control group at 40* magnification, **(B-c)** Lungs of the ALI model group after 24 hours of LPS exposure at 20* magnification, **(B-d)** Lungs of the ALI model group after 24 hours of LPS exposure at 40* magnification. Among them, the red arrow represents granulocyte infiltration, the blue arrow represents alveolar septum, the black arrow represents eosinophil infiltration, the yellow arrow represents lymphocyte infiltration, the orange arrow represents epithelial cell degeneration, and the brown arrow represents edema. **(C)** Levels of TNF-*α*, IL-6, IL-1*β*, IFN-*γ*, PGE2, MDA, and GSH in serum of the control group and ALI model group rats after 24 hours of LPS attack. The value of MDA is the product of its actual content multiplied by 100 times. **(D)** Levels of lungs, spleen, and thymus coefficient of the control group and ALI model group rats. n = 8 per group. Difference between the control group and ALI model group rats were assessed by one-way ANOVA, compared with the control group, the ALI model group *p < 0.05, **p < 0.01, ***p < 0.001, ****p < 0.0001.

Compared with those of the control group rats, the red blood cell count, white blood cell count, neutrophil count, and hemoglobin in serum were significantly increased, while platelet count in serum were significantly reduced in the model group (Fig 2A). As shown in Fig 2B, ALI was caused by an increase in the contents of TNF-*α*, IL-6, IL-1*β*, IFN-*γ*, PGE2, and MDA, and a decrease in the content of GSH. In accordance with the observation of the lung histopathological sections (Fig 2C), there was numerous infiltration of granulocytes and thickening in the alveolar wall of the model group, and widening of the alveolar septum could be widely seen. Additionally, a small amount of edema could be seen around the blood vessels, with loose arrangement of connective tissue and many inflammatory cell infiltration mainly consisting of lymphocytes and granulocytes could be observed. Besides, the decrease in thymus coefficient and pathological enlargement of spleen and lung tissue were detected (Fig 2D). These results indicated that the LPS induced ALI model in rats has been successfully replicated.

### 3.2. Biomarkers of ALI model

**Fig 3:**
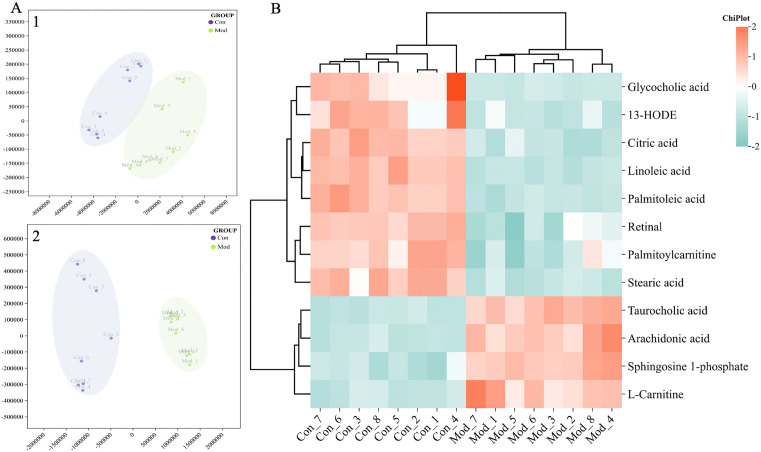
Multivariate data statistical analysis and potential effect markers of ALI model on serum metabolic profile. **(A)** PCA plot of serum metabolism of control and ALI model group. (A-1) PCA plot of serum metabolism of control and model group in the ESI^+^ mode. (A-2) PCA plot of serum metabolism of control and model group in the ESI^−^ mode. **(B)** Heatmap of the content changes in the potential serum ALI biomarkers of the control group and ALI model group.

The serum samples of control group and model group rats were analysed to exhume the biomarkers of ALI model. The results showed the metabolic profile of rats in the model group was obviously distinguished from that in the control group (S1 Fig). The PCA score plots in Fig 3A showed that there was a clear trend of separation between the control group and the model group. Finally, a total of 12 potential serum metabolite biomarkers related to the ALI rat model were identified (Table 1). The difference in biomarker content between the control group and the ALI model group was clearly demonstrated using a heatmap (Fig 3B).

**Table 1:** Statistical analysis of serum biomarkers in ALI model.

No.	Rt	m/z	Ion mode	Formula	Compound	FDR *p*-Value	Trend
1	0.84	162.1126	ESI^+^	C_7_H_15_NO_3_	L-Carnitine	1.35E-12	↑
2	0.94	191.0188	ESI^-^	C_6_H_8_O_7_	Citric acid	1.76E-12	↓
3	2.71	514.2840	ESI^-^	C_26_H_45_NO_7_S	Taurocholic acid	9.01E-06	↑
4	2.92	464.3014	ESI^-^	C_26_H_43_NO_6_	Glycocholic acid	7.93E-06	↓
5	3.87	378.2410	ESI ^+ /-^	C_18_H_38_NO_5_P	Sphingosine 1-phosphate	3.49E-15	↑
6	4.27	253.2166	ESI^-^	C_16_H_30_O_2_	Palmitoleic acid	0.001261	↓
7	4.51	303.2323	ESI^-^	C_20_H_32_O_2_	Arachidonic acid	6.07E-08	↑
8	4.67	279.2324	ESI^-^	C_18_H_32_O_2_	Linoleic acid	4.07E-07	↓
9	4.74	400.3433	ESI^+^	C_23_H_45_NO_4_	Palmitoylcarnitine	1.15E-08	↓
10	5.42	295.2273	ESI^-^	C_18_H_32_O_3_	13-HODE	0.000102	↓
11	5.91	285.2225	ESI^+^	C_20_H_28_O	Retinal	2.59E-07	↓
12	6.34	283.2637	ESI^-^	C_18_H_36_O_2_	Stearic acid	4.07E-13	↓

Note: **↑** indicates an increase in biomarker content, and ↓ indicates a decrease in biomarker content. 13-HODE is 13-Hydroxyoctadecadienoic acid.

**Fig 4:**
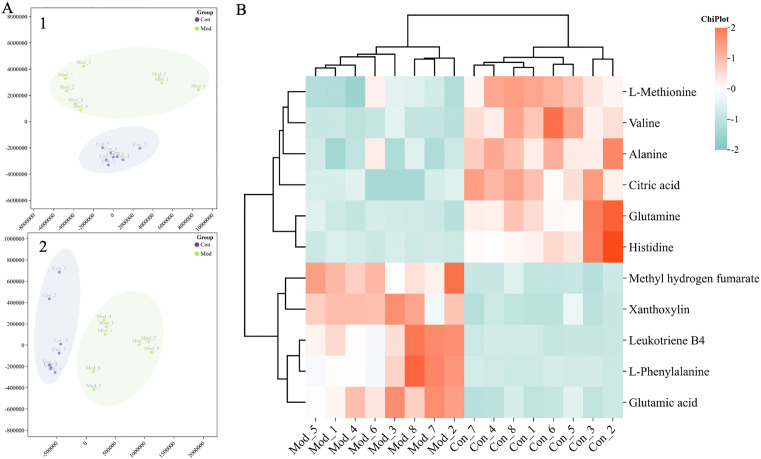
Multivariate data statistical analysis and potential effect markers of ALI model on urine metabolic profile. **(A)** PCA plot of urine metabolism of control and ALI model group. (A-1) PCA plot of urine metabolism of control and model group in the ESI^+^ mode. (A-2) PCA plot of urine metabolism of control and model group in the ESI^−^ mode. **(B)** Heatmap of the content changes in the potential urine ALI biomarkers of the control group and ALI model group.

Urine samples were tested according to the above method to obtain the urine metabolic profile (S2 Fig), and PCA analyses were also performed to identify urine biomarkers (Fig 4A). Ultimately, 11 ALI urine biomarkers were obtained and visualized for content changes (Table 2, Fig 4B).

**Table 2:** Statistical analysis of urine biomarkers in ALI model.

No.	Rt	m/z	Ion mode	Formula	Compound	FDR *p*-Value	Trend
1	0.67	118.0865	ESI^+^	C_5_H_11_NO_2_	Valine	2.85E-16	↓
2	1.30	215.0176	ESI^+^	C_6_H_8_O_7_	Citric acid	1.71E-06	↓
3	1.71	129.0183	ESI^-^	C_5_H_6_O_4_	Methyl hydrogen fumarate	4.79E-12	↑
4	1.99	194.0484	ESI^-^	C_5_H_11_NO_2_S	L-Methionine	0.000361269	↓
5	2.54	164.0705	ESI^-^	C_9_H_11_NO_2_	L-Phenylalanine	2.19E-07	↑
6	2.87	90.0549	ESI^+^	C_3_H_7_NO_2_	Alanine	6.37E-08	↓
7	4.07	309.1246	ESI^-^	C_6_H_9_N_3_O_2_	Histidine	3.13E-11	↓
8	4.65	393.1538	ESI^+^	C_10_H_12_O_4_	Xanthoxylin	4.99E-15	↑
9	4.95	381.2270	ESI^-^	C_20_H_32_O_4_	Leukotriene B4	7.13E-13	↑
10	7.15	295.1104	ESI^+^	C_5_H_9_NO_4_	Glutamic acid	1.81E-17	↑
11	7.48	291.1281	ESI^-^	C_5_H_10_N_2_O_3_	Glutamine	6.40E-19	↓

Note: **↑** indicates an increase in biomarker content, and ↓ indicates a decrease in biomarker content.

### 3.3. JFG alleviated lung pathological damage in ALI by restoring hematological parameters and reducing inflammatory cytokines

**Fig 5:**
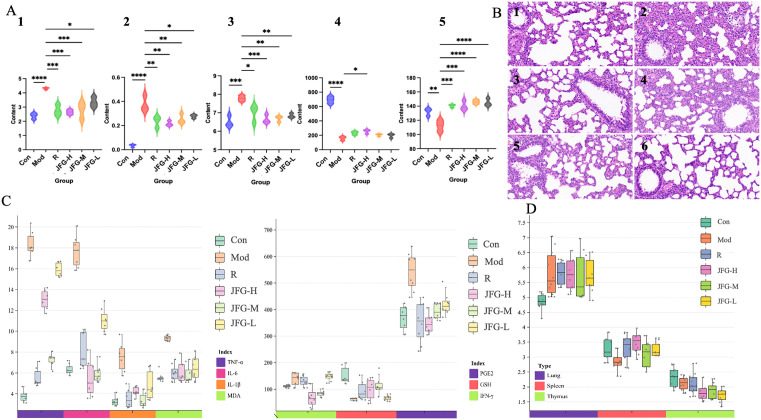
The preventive effect of JFG on ALI induced by LPS. **(A)** Number of platelets, white blood cells, and neutrophils in the blood after 24 hours of LPS attack. **(A-a)** White blood cell count, **(A-b)** Neutrophil count, **(A-c)** Red blood cell count, **(A-d)** Platelet count, **(A-e)** Hemoglobin. **(B)** Hematoxylin and eosin **(H&E)**-stained lung sections of rats at 24 hours after LPS challenge, the magnification of the microscope is × 100. **(B-a)** Lungs of blank control group, **(B-b)** Lungs of the model group after 24 hours of LPS exposure, **(B-c)** Lungs of the DXMS group after 24 hours of LPS exposure, **(B-d)** Lungs of the JFG-H group after 24 hours of LPS exposure, **(B-e)** Lungs of the JFG-M group after 24 hours of LPS exposure, **(B-f)** Lungs of the JFG-L group after 24 hours of LPS exposure. **(C)** Levels of TNF-*α*, IL-6, IL-1*β*, IFN-*γ*, PGE2, MDA, and GSH in serum after 24 hours of LPS attack. The value of MDA is the product of its actual content multiplied by 100 times. **(D)** Levels of lungs, spleen, and thymus coefficient of rats. n = 8 per group. Difference between the other group and ALI model group rats were assessed by one-way ANOVA, compared with the ALI model group, *p < 0.05, **p < 0.01, ***p < 0.001, ****p < 0.00001.

JFG-H, JFG-M, and JFG-L groups significantly corrected the abnormal content levels of ALI-related biochemical index, incluidng red blood cell count, white blood cell count, neutrophil count, hemoglobin, and platelet count (Fig 5A). Simultaneously improving edema and inflammatory damage in lung tissue (Fig 5B), and reversing the levels of a series of inflammatory related substances in serum, including a decrease in TNF-*α*, IL-6, IL-1*β*, IFN-*γ*, PGE2, and MDA, and an increase in GSH (Fig 5C). In addition, the tissues with pathological changes were restored (Fig 5D). Remarkably, the therapeutic effect of JFG was close to that of the positive control group.

### 3.4. JFG callback the trajectory of AA, LA, L-phenylalanine, and leukotriene B4 in ALI rats

**Fig 6:**
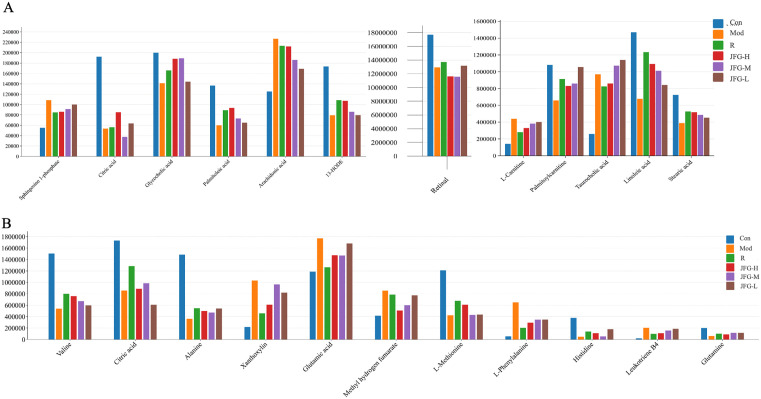
Potential therapeutic effect biomarkers of JFG on ALI. **(A)** Changes in the content of potential effect markers of JFG on ALI in serum. **(B)** Changes in the content of potential effect markers of JFG on ALI in urine.

JFG could significantly ameliorate the abnormal metabolic profile of the ALI rats. elevated sphingosine 1-phosphate and AA, and reduced LA and citric acid in the serum of ALI rats were reversed (Fig 6A). Similarly, JFG profoundly altered the urine metabolomic characteristics of ALI rats, including xanthoxylin and leukotriene B4 with increased content, and citric acid and L-phenylalanine with decreased content (Fig 6B).

### 3.5. JFG regulated LA metabolism, AA metabolism, and phenylalanine, tyrosine and tryptophan biosynthesis

**Fig 7:**
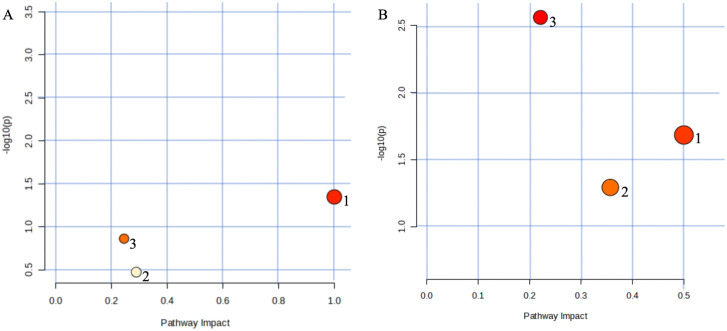
Metabolic pathway analysis of JFG therapeutic effect *in vivo.* **(A)** Metabolic pathway analysis of JFG therapeutic effect on ALI rats based on serum samples (1: Linoleic acid metabolism, 2: Arachidonic acid metabolism, 3: Retinol metabolism). **(B)** Metabolic pathway analysis of JFG therapeutic effect on ALI rats based on urine samples (1: Phenylalanine, tyrosine and tryptophan biosynthesis, 2: Phenylalanine metabolism, 3: Histidine metabolism).

The pathway analysis was performed on 12 serum and 11 urine effect markers in the JFG therapeutic group through the MetaboAnalyst website. 3 serum metabolic pathways were obtained using the impact value > 0.2 as a screening parameter, including LA metabolism, AA metabolism, and retinol metabolism (S1 Table, Fig 7A). Equally, 3 urine metabolic pathways were obtained, including phenylalanine, tyrosine and tryptophan biosynthesis, phenylalanine metabolism, and histidine metabolism (S2 Table, Fig 7B).

**Fig 8:**
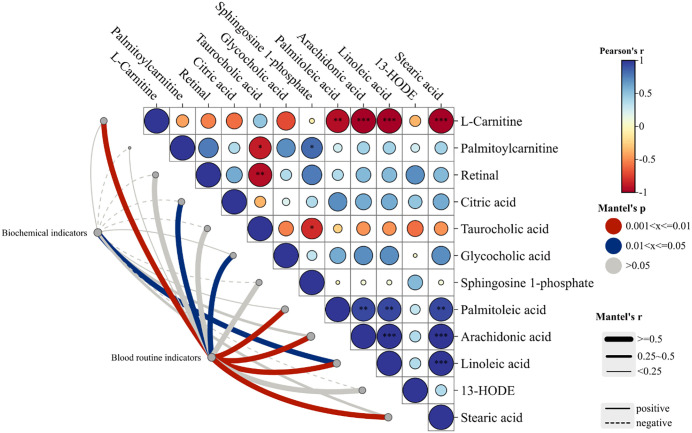
Correlation analysis between biochemical indicators and serum effect biomarkers. The blood routine indicators include white blood cell count, neutrophil count, red blood cell count, platelet count, ane hemoglobin. The biochemical indicators include levels of TNF-*α*, IL-6, IL-1*β*, IFN-*γ*, PGE2, MDA, and GSH in serum.

The correlation between biochemical indicators and effect markers was analyzed to identify the major therapeutic effect biomarkers of JFG against ALI. Obviously, L-carnitine, AA, LA, palmitoleic acid, citric acid, glycocholic acid, and stearic acid were confirmed as major serum effect markers of JFG (Fig 8), especially the regulation of LA, AA, palmitoleic acid, L-carnitine, and stearic acid occupied a dominant position.

**Fig 9:**
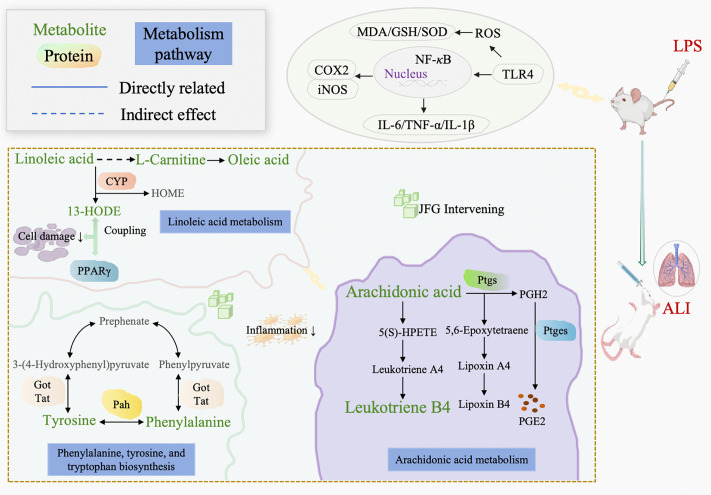
The association mechanism diagram of the metabolic pathways of therapeutic effect of JFG on ALI. Green vocabulary is potential effect biomarkers *in vivo*; red vocabulary is key protein associated with effect biomarkers and their metabolites, gray vocabulary is the metabolites of effect biomarkers, vocabulary with a blue background is metabolic pathway, the solid line with arrows represents the generation or promotion relationship, while the dashed line with arrows represents the competition or inhibition relationship.

These 5 key serum effect biomarkers mostly belonged to LA metabolism and AA metabolism. Additionally, through urine metabolomics analysis, we found that the therapeutic effect of JFG on ALI rats was most closely related to the phenylalanine, tyrosine and tryptophan biosynthesis pathway. Accordingly, we integrated and drawn a mechanism diagram of the metabolic pathways of therapeutic effect of JFG on ALI basis on the HMDB and the KEGG database (Fig 9).

## 4. Discussion

Due to its complex, acute, and inflammatory characteristics, ALI can cause a range of respiratory symptoms and cardiovascular diseases, seriously affecting the quality of patient’s life [41]. At present, although progress has been made in understanding the pathogenesis of ALI, its treatment is still limited by systemic side effects and incomplete efficacy. Therefore, biological targeted drugs and multi-target regulation therapies in TCM are highly anticipated. JFG is one of the classic prescriptions for treating ALI in TCM theory and clinical experience, but its pharmacological mechanism is still unclear. This study used LPS-induced ALI model to systematically evaluate the intervention effect of JFG on ALI by analyzing lung pathological status, inflammation assessment indicators, immune organ status, and metabolomics. It was found that JFG can affect LA metabolism, phenylalanine, tyrosine, and tryptophan biosynthesis, as well as AA metabolic pathways in ALI rats by the callback of potential effector biomarkers including LA, AA, palmitoleic acid, L-carnitine, L-phenylalanine, and stearic acid, achieving multi-target intervention on inflammation, oxidative stress, and metabolic disorders in ALI. This addresses two key gaps in current inadequate treatment of ALI and insufficient scientific validation of JFG, emphasizing the potential of JFG as a clinical solution for ALI.

TNF-*α* can damage endothelial cells and stimulate the release of other inflammatory mediators [42], IL-1*β* and IL-6 are important inflammatory mediators that can induce neutrophils aggregation in the lungs, thereby exacerbating the inflammatory response [43]. After JFG, the abnormal elevation of TNF-*α*, IL-6, and IL-1*β* caused by LPS was reversed. Besides, the significant increase in white blood cell and neutrophil counts, and the significant decrease in platelet counts in the blood, are the main characteristics of acute bleeding and pulmonary infections [44,45]. JFG also increased platelet levels and decreased white blood cell and neutrophil levels, alleviating the inflammatory response when exposed to LPS attack. Additionally, our study found that JFG effectively associated with changes in the AA metabolic pathway, LA metabolic pathway, as well as phenylalanine, tyrosine, and tryptophan biosynthesis pathways during the intervention process, which are closely related to inflammation, energy metabolism homeostasis, and oxidative stress regulation.

AA is a precursor for synthesizing pro-inflammatory eicosanoids compounds, including prostaglandins, thromboxanes, and leukotrienes (LT) [46]. In LPS-infected ALI, these mediators increase vascular permeability, promote bronchoconstriction, and exacerbate lung inflammation, their elevated levels can explain the severe damage to the lungs during infection [47,48]. These lipid mediators further activate pro-inflammatory signaling pathways, exacerbating the progression of ALI [49,50]. Importantly, JFG administration significantly reduced the levels of key inflammatory mediators AA, LTB4, and PGE2, particularly LTB4. The marked reduction of LTB4 in the JFG group directly explains the observed attenuation of neutrophil infiltration in lung tissue, providing a mechanistic link between the metabolic data and the histopathological improvement.

JFG synergistically enhances the anti-inflammatory effect by regulating fatty acid metabolism. During ALI, inflammation and oxidative stress can disrupt normal fatty acid homeostasis, leading to impaired fatty acid oxidation and increased lipid droplet accumulation [51,52]. JFG treatment normalizes metabolic disorders of key fatty acid biomarkers such as LA, oleic acid, 13-HODE, and palmitic acid effectively, restoring lung homeostasis. In addition, ALI is also characterized by oxidative stress imbalance. LA treatment has been shown to increase the levels of antioxidants such as superoxide dismutase and GSH, while reducing the production of lipid peroxidation marker MDA [53,54]. By reversing the disruption of LA metabolic pathway during ALI, JFG reduced MDA levels in rat blood, increased GSH content, and reduced oxidative stress damage, providing protection for lung tissue under LPS attack. The dual regulation of AA and LA metabolic pathways by JFG represents a new therapeutic strategy that simultaneously addresses the inflammatory and metabolic components of ALI, indicating that early JFG intervention may become an effective measure to prevent severe ALI progression and improve clinical outcomes, which may provide important research directions for future lung protection. We observed an increase in pro-inflammatory mediators of AA and LA in the ALI model, further demonstrating that JFG can partially reverse this imbalance, suggesting that JFG inhibits the pro-inflammatory LOX and COX pathways by associating with changes in AA/LA metabolism. In subsequent experiments, we will validate our hypothesis through enzyme activity assays, gene sequencing, and other methods.

The interplay between the biosynthetic pathways of phenylalanine, tyrosine, andtryptophan and the treatment of ALl is of significant importance. Tryptophan can be metabolized into bioactive substances that regulate inflammation, and may also participate in the production of reactive oxygen species, which is a key process involved in ALI [55,56]. By significantly rebalancing the tryptophan-kynurenine pathway, JFG reduces the production of pro-inflammatory cytokines while enhancing cellular antioxidant capacity and immune function. Tyrosine is a precursor of catecholamines including dopamine, norepinephrine, and adrenaline [57]. Through regulating phenylalanine metabolism and restoring tyrosine homeostasis, JFG may disrupt the interplay between oxidative stress and inflammation processes, alleviating LPS-induced lung dysfunction. These results indicate that JFG can simultaneously target multiple interrelated amino acid metabolic pathways, elucidating the important mechanism of JFG treatment for ALI while addressing the current lack of reliable biomarkers for evaluating the efficacy of TCM.

In summary, JFG is a TCM compound preparation that has a significant protective effect on ALI, and the key mechanism of action involves reducing inflammatory reactions, which is a key pathological process of ALI, as well as the combined effects of oxidative stress regulation and immune function enhancement. These also confirm the unique properties of TCM formulas in disease intervention and treatment. However, our research also has inherent limitations. Although we observed that LA metabolism, phenylalanine, tyrosine, tryptophan biosynthesis, and AA metabolism were regulated and inflammatory factors were inhibited in ALI rats after receiving JFG, we concluded that JFG has the ability to inhibit inflammation and promote immunity. Nevertheless, existing data is limited, and a single preclinical animal model of ALI cannot fully simulate the complexity and heterogeneity of human ALI/ARDS, and there is a lack of pharmacokinetic data to link the herbal components in JFG with observed metabolic changes. We will conduct more extensive experimental studies and validations in the future to explore the therapeutic mechanism of JFG in depth and ultimately conduct clinical trials to evaluate its translational potential in humans. Additionally, given the emerging link between ecological imbalance and the severity of ALI, the exact contribution of the gut microbiota-short chain fatty acid axis to JFG intervention in ALI is worth studying. These are not only crucial for elucidating the complex pharmacological mechanisms of JFG in treating ALI, but also help to understand and explore its potential value in preventing and treating additional inflammatory diseases.

## 5. Conclusion

JFG precisely regulates the levels of endogenous metabolites such as LA, AA, palmitoleic acid, L-carnitine, L-phenylalanine, and stearic acid in ALI, thereby associating with changes in the LA metabolic pathway, phenylalanine, tyrosine, and tryptophan biosynthesis, as well as the AA metabolic pathway, ultimately reshaping the balance of inflammation and oxidative stress, and exerting a significant intervention effect. Looking forward, the translational pathway for JFG involves traversing key pre-clinical and clinical paradigms. Immediately, pharmacokinetic studies are required to define its optimal therapeutic window and active components. Subsequently, clinically translating these findings to address the challenges of patient stratification, endpoint selection, and standardization of the herbal formulation. Despite the challenges, this work provides a robust scientific foundation and a clear roadmap for developing JFG as a promising therapeutic strategy for a condition in urgent need of new treatment options.

## Supporting information

S1 Table Metabolic pathway analysis of JBP therapeutic effect in serum. (PDF)

S2 Table Metabolic pathway analysis of JBP therapeutic effect in urine. (PDF)

S1 Fig Metabolic profile of JBP therapeutic effect in serum. (PDF)

S2 Fig Metabolic profile of JBP therapeutic effect in urine. (PDF)

## Abbreviation:

AA: arachidonic acid
ALI: acute lung injury
ARDS: acute respiratory distress syndrome
JFG: Jingfang Granules
LA: linoleic acid
LPS: lipopolysaccharide
LT: leukotrienes
QC: quality control
TCM: traditional Chinese medicine
