# Supplementary material for: Jingfang granules inhibiting LPS-induced acute lung injury via regulating linoleic acid and arachidonic acid metabolism pathway
Source: PLoS One. 2026 Jan 16;21(1):e0340858. doi: 10.1371/journal.pone.0340858 (PMC12810783; doi:10.1371/journal.pone.0340858)
Supplement: S1 Table — (PDF) [file pone.0340858.s001.pdf]

Supplementary Table 1. Metabolic pathway analysis of JBP therapeutic effect in serum

Pathway	Total	Expected	Hits	Raw p	Holm adjust	FDR	Impact
Linoleic acid metabolism	5	0.0323	1	0.031926	1	0.89084	1
Arachidonic acid metabolism	44	0.28424	1	0.25114	1	1	0.2893
Retinol metabolism	16	0.10336	1	0.098957	1	1	0.24551
Citrate cycle (TCA cycle)	20	0.1292	1	0.12228	1	1	0.09038
Glyoxylate and dicarboxylate metabolism	32	0.20672	1	0.18901	1	1	0.08
Primary bile acid biosynthesis	46	0.29716	2	0.033406	1	0.89084	0.0457
Sphingolipid metabolism	32	0.20672	1	0.18901	1	1	0.01563
