# Supplementary material for: Jingfang granules inhibiting LPS-induced acute lung injury via regulating linoleic acid and arachidonic acid metabolism pathway
Source: PLoS One. 2026 Jan 16;21(1):e0340858. doi: 10.1371/journal.pone.0340858 (PMC12810783; doi:10.1371/journal.pone.0340858)
Supplement: S2 Table — (PDF) [file pone.0340858.s002.pdf]

Supplementary Table 2. Metabolic pathway analysis of JBP therapeutic effect in urine

Pathway	Total	Expected	Hits	Raw p	Holm adjust	FDR	Impact
Phenylalanine, tyrosine and tryptophan biosynthesis	4	0.020888	1	0.02075	1	0.4149	0.5
Phenylalanine metabolism	10	0.052219	1	0.05116	1	0.5846	0.35714
Histidine metabolism	16	0.083551	2	0.00276	0.2210	0.2201	0.22131
Alanine, aspartate and glutamate metabolism	28	0.14621	2	0.00843	0.6660	0.2919	0.19712
Arginine biosynthesis	14	0.073107	1	0.07097	1	0.6069	0.11675
Cysteine and methionine metabolism	33	0.17232	1	0.16022	1	0.8011	0.10446
Citrate cycle (TCA cycle)	20	0.10444	1	0.10001	1	0.6447	0.09038
Glyoxylate and dicarboxylate metabolism	32	0.1671	2	0.01095	0.8538	0.2919	0.03175
Glutathione metabolism	28	0.14621	1	0.13749	1	0.7857	0.01966
Arachidonic acid metabolism	44	0.22977	1	0.20838	1	0.87738	0.01528
