# Supplementary figures and images for: Jingfang granules inhibiting LPS-induced acute lung injury via regulating linoleic acid and arachidonic acid metabolism pathway
Source: PLoS One. 2026 Jan 16;21(1):e0340858. doi: 10.1371/journal.pone.0340858 (PMC12810783; doi:10.1371/journal.pone.0340858)

Supplementary Figure 1. Metabolic profile of JBP therapeutic effect in serum

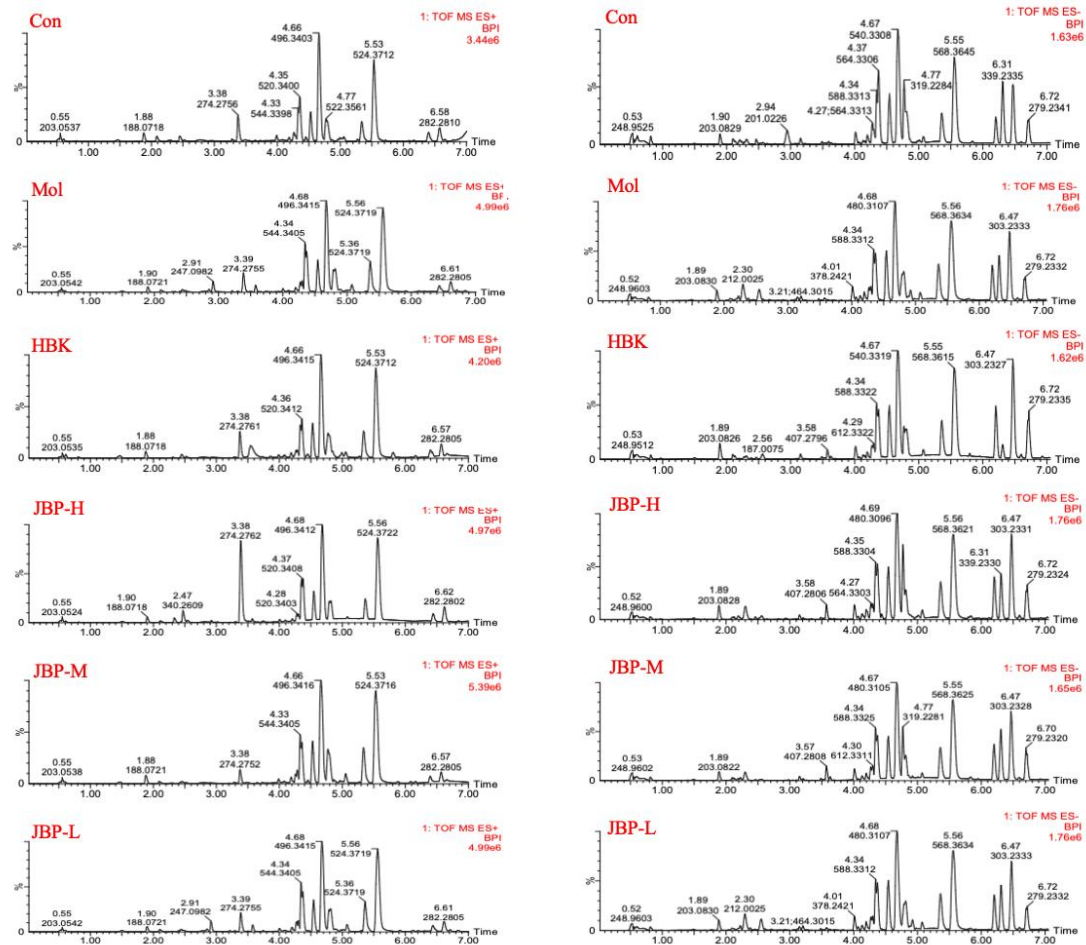

Supplement: S1 Fig — (PDF) [file pone.0340858.s003.pdf]

Supplementary Figure 2. Metabolic profile of JBP therapeutic effect in urine

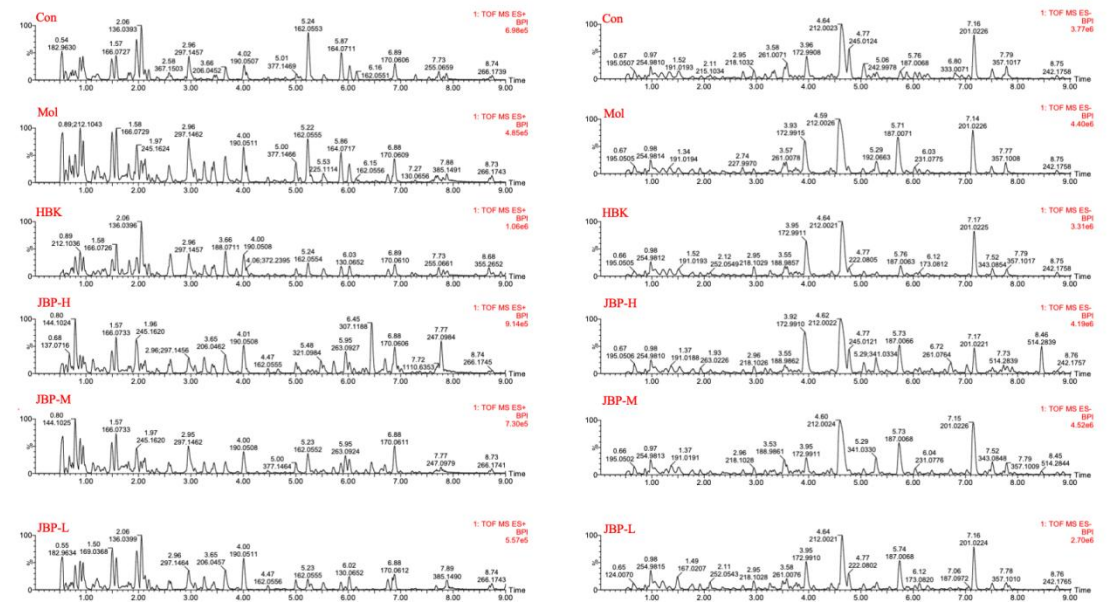

Supplement: S2 Fig — (PDF) [file pone.0340858.s004.pdf]
